# Examining the Distribution of Strength across the Thickness of Reinforced Concrete Elements Subject to Sulphate Corrosion Using the Ultrasonic Method

**DOI:** 10.3390/ma12162519

**Published:** 2019-08-07

**Authors:** Bohdan Stawiski, Tomasz Kania

**Affiliations:** 1 Faculty of Environmental Engineering and Geodesy, Wrocław University of Environmental and Life Sciences, pl. Grunwaldzki 24, 50-363 Wrocław, Poland; 2 Faculty of Civil Engineering, Wrocław University of Science and Technology, Wybrzeże Wyspiańskiego 27, 50-370 Wrocław, Poland

**Keywords:** concrete elements, concrete strength, reinforced concrete tanks, concrete corrosion, sulphate corrosion, ultrasound tests

## Abstract

Sulphate corrosion of concrete is a complex chemical and physical process that leads to the destruction of construction elements. Degradation of concrete results from the transportation of sulphate compounds through the pores of exposed elements and their chemical reactions with cementitious material. Sulphate corrosion can develop in all kind of structures exposed to the corrosive environment. The mechanism of the chemical reactions of sulphate ions with concrete compounds is well known and described. Furthermore, the dependence of the compressive strength of standard cubic samples on the duration of their exposure in the sulphate corrosion environment has been described. However, strength tests on standard samples presented in the scientific literature do not provide an answer to the question regarding the measurement methodology and actual distribution of compressive strength in cross-section of reinforced concrete structures exposed to sulphate ions. Since it is difficult to find any description of this type of test in the literature, the authors undertook to conduct them. The ultrasonic method using exponential heads with spot surface of contact with the material was chosen for the measurements of concrete strength in close cross-sections parallel to the corroded surface. The test was performed on samples taken from compartments of a reinforced concrete tank after five years of operation in a corrosive environment. Test measurements showed heterogeneity of strength across the entire thickness of the tested elements. It was determined that the strength of the elements in internal cross-sections of the structure was up to 80% higher than the initial strength. A drop in the mechanical properties of concrete was observed only in the close zone near the exposed surface.

## 1. Introduction

There are considerable quantities of effluents generated in chemical laboratories with varied pH, which is the measure of acidity or alkalinity of an aqueous solution. Neutral solutions have a pH of approximately 7.0. Neutralisation of effluents is performed by mixing acidic and alkaline compounds (if their compositions allow it) and by adding acidic or alkaline reagents. This takes place in various types of tanks. For neutralisation of laboratory effluents, reinforced concrete tanks are also used. The ratio of acidity and alkalinity is a critical factor in the chemistry of concrete [1]. The components of concrete are cement, aggregates, and water. Cement has a very alkaline pH, in order to bind all the components, it is important for it to remain near a pH of 12 [2]. In contact with effluents, concrete corrodes. Therefore, it should be characterized by the proper strength and tightness, and should be protected from the aggressive environment by the proper lining [3].

In the literature, one may encounter the opinion that after 1989 the quality of reinforced concrete structures in Poland improved radically [4], but problems with the materials, workmanship and design still exist, as can be seen in the latest research on Polish concrete structures [5,6]. The neutralisation tank presented later was made in 2012 and became corroded, which indicates that concrete insufficiently protected from corrosion will require repair, which should be preceded by a good evaluation of the condition of the damaged structure.

Concrete corrosion not only affects laboratory tanks but develops in all kind of structures. Concrete durability is the constant subject of challenges in the fields of science, design and workmanship [5,6]. As a consequence of concrete structures’ exposure to corrosive environments, various substances are being transported into the concrete, causing its expansion, cracking, and strength degradation. Among the most destructive of the numerous corrosive substances are the sulphates [7].

Recent studies on sulphate corrosion of concrete are mainly focused on the mechanism of the chemical reactions of sulphate ions with the concrete compounds [8,9,10,11,12,13,14,15,16] and the distribution of strength over time of cubic samples stored in sulphate solutions. The corrosive reactions of sulphates in concrete have been well studied and evaluated [17,18,19,20]. The deterioration of concrete strength under sulphate corrosion is an essential basis for the prediction of concrete performance and durability. Existing studies indicate that sulphate ions in the environment chemically react with the internal composition of concrete by entering into the concrete through diffusion, convection, capillary adsorption, and other processes to generate expansive products such as ettringite, gypsum [17,18,19,20], and sodium sulphate crystals when concrete is corroded by sulphate solution in a dry-wet cycle. The expansive products continuously fill the internal pores of concrete, making the concrete more compact with improved concrete strength before deterioration. Ions of sulphuric acid react with the cement compounds e.g., according to Equations (1), (2) and (3) [7]:

H_2_SO_4_ + CaO ∙ SiO_2_ ∙ 2H_2_O → CaSO_4_ + Si(OH)_4_ + H_2_O (1)

H_2_SO_4_ + CaCO_3_ → CaSO_4_ + H_2_CO_3_ (2)

H_2_SO_4_ + Ca(OH)_2_ → CaSO_4_ + 2H_2_O (3)

The formation of gypsum leads to an increase in volume of approximately 124% in comparison with Ca(OH)_2_, the main reactant of the process [17,18]. Gypsum stone, as the product of reactions (1), (2) and (3) reacts further with tricalcium aluminates (C3A) or hydrated calcium sulfoaluminate (monosulphate) to form the final chemical product Candlot’s salt (ettringite), e.g., according to Reaction (4) [18]:

3CaO ∙ Al_2_O_3_ + 3(CaSO_4_ ∙ 2H_2_O) + 26H_2_O → 3CaO ∙ Al_2_O_3_ ∙ 3CaSO_4_ ∙ 32H_2_O (4)

Formation of Candlot’s salt is associated with volume expansion from 230% [9] to 820% [10]. According to [21] the following prerequisites must be reached for Candlot’s salt crystallization leading to the concrete expansion:

- The volume of Candlot’s salt must exceed some threshold value which depends on the capillary porosity of concrete,
- Only Candlot’s salt formed after the hydration of cement leads to expansion,
- Candlot’s salt must be formed at the boundaries of solid phases of concrete.

Candlot’s salt crystallization pressure depends on the sulphate concentration and can reach the value of 35 N/mm^2^ with sulphate concentration of 350 mol/m^3^ [18].

The exemplary relationships of sulphate corrosion with strength of concrete samples immersed in sulphate solution have been established and described [21,22,23]. Zhou et al. in [21] stated that the compressive strength of cubic samples conditioned in dry-wet cycles in sulphate solution shows the rise period and decline area. The strength of concrete samples reached its peak at the 60th day of corrosion and increased by ∼6.4% on the basis of its initial strength. With the increase in degradation period, the strength of concrete decreased continuously. The compressive strength decreased by ∼4.4%, 18%, and 43.1% after 90, 120, and 150 days of corrosion. This research was done under laboratory conditions on standard cubic samples with a side length of 150 mm tested on a strength machine. Shi and Wang in [22] stated that strength of concrete samples conditioned in dry-wet cycles in 15% sodium sulphate solution reached its peak at the 15th day of corrosion and increased by 29% on the basis of its initial value. Du et al. in [23] tested the C25 concrete mixed with 20% fly ash placed in a sodium sulfate solution (20%) for the full-soaking corrosion test. Samples reached peak of strength at the 100th day of corrosion and increased by 10.6% on the basis of its initial value. Due to the methodology of these tests, the distribution of strength in individual cross-sections of samples which had been previously exposed to the corrosive environment was not determined experimentally.

Laboratory tests of sulphate attack on concrete materials that are based on submerging the specimens in sulphate solution and then measuring physical properties, such as strength, are effectively collecting all of these mechanisms into a single test. The result of such research is the characterization of a particular concrete sample’s performance under specific, laboratory conditions. If the field conditions are variable, the performance of the concrete can also be different. Concrete compressive strength in structures is designed to withstand the designed forces. The durability of the structure depends on the fulfilment of the limit condition of concrete strength in the section that works in the state of compression. The question arises what is the compressive strength distribution in various cross sections parallel to the surface of a reinforced concrete structure under sulphate corrosion? Since tests of such type have not been performed so far, it is difficult to find appropriate literature references regarding possible methodology or results concerning the distribution of compressive strength across the thickness of reinforced concrete elements exposed to sulphate corrosion.

This paper presents research on the concrete samples taken from a concrete neutralization tank after five years of storing chemical effluents with sulphate compounds. The main purpose of the experimental tests was to determine the compressive strength of concrete in various cross sections parallel to the corroded surface. It has been stated that compressive strength of concrete subjected to the gaseous aggressive, sulphuric environment is variable across its thickness. Values have shown the increase in strength in the internal, exposed cross sections of the walls.

## 2. Materials and Methods

### 2.1. Materials

In the described case study, a neutralisation tank in a building with numerous chemical laboratories was examined. The described tank is being used for the neutralization of liquid wastes from a research program in the field of pharmaceutical production. The chemical composition of the effluents is variable over time and depends on the actual research program in the medical laboratory. A research object selected in this way allows assessment of the corrosion condition of concrete under unplanned conditions and evaluation of the change in the condition of samples of material taken from a real object in operation. The tank was designed as a reinforced concrete box, internally divided into three chambers. The tank was constructed of reinforced, water resistant concrete class C25/30, W8. The tank was made using monolithic technology and its walls were formed in the built-in (vertical) position.

The designer anticipated protecting the concrete using epoxy chemical-resistant lining. The reinforced concrete ceiling above the chambers was made on folded sheets (as composite stay-in-place formwork). Access to each of the chambers is via a manhole covered with a stainless steel lid. The ceiling slab across its thickness in the locations of the manholes was probably not protected from corrosion because during tests there were not any traces of any layer after five years of using the tank.

**Figure 1:**
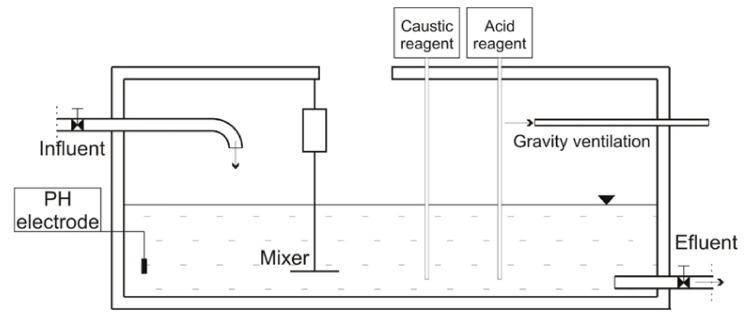
Method diagram of the neutralisation process in the concrete tank.

The described neutralisation tank operates in the batch mode. The chemical composition of the effluents that are being neutralised is variable over time and depends on the actual research and production program in the pharmaceutical laboratory. Effluents are pumped into the chamber where an electrode measures their pH. On that basis effluents are evaluated in terms of their compliance with the set point (neutral pH value). If the pH value is out of range, chemical pumps inject an acid or caustic reagent solution as required to bring the effluent to the correct level. The agitator keep the contents of the tank mixed, so the pH probe is always measuring a representative sample of the effluent and the added reagents are quickly distributed within the tank. For the caustic reagent solutions of caustic soda (NaOH, Chempur, Piekary Slaskie, Poland) are used. For the acid reagent a solution of sulphuric acid (H_2_SO_4_, Chempur, Piekary Slaskie, Poland) is used. A schematic method diagram of the described neutralization in the tank is shown on the Figure 1.

After neutralisation of effluents down to neutrality they are pumped out to the sewage system. The neutralisation process is accompanied by emission of gases which should be discharged outside, preferably using gravity ventilation or mechanical ventilation resistant to the aggressive environment. Gravity ventilation should have large cross-sections of ducts and small deviations from the vertical. Traditionally, they are openings made of brick 140 x 140 mm or round ϕ 150 mm. In the examined tank, the ‘ventilation’ was made of PVC pipes (Wiplast, Twardogora, Poland), diameter 50 mm, which were laid horizontally on the tank for a distance of approximately 2 m. These two parameters are sufficient to indicate its lack of effectiveness.

The tank became a natural experimental ground with respect to the effects of long-term exposure to liquid and gaseous aggressive environments on concrete, the internal surfaces of which were protected from liquid effluents using an asphalt rubber coating (probably made of Dysperbit) instead of the designed epoxy lining. No protective coating was applied for contact with the gaseous environment.

**Figure 2:**
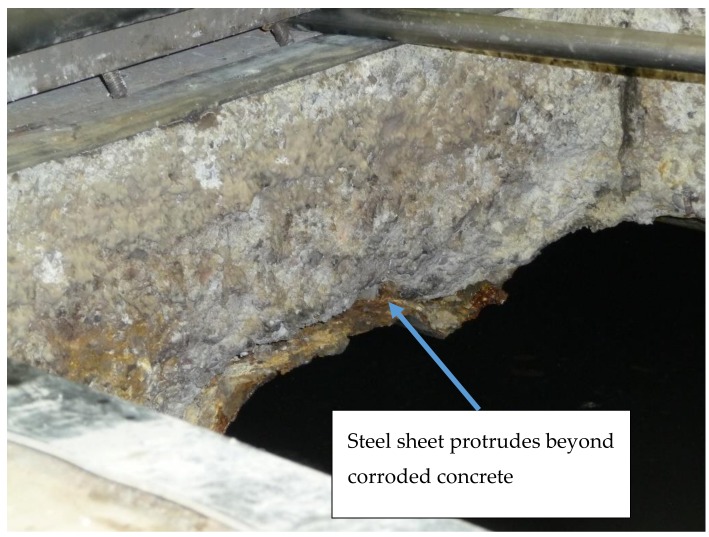
The folded sheet corroded more slowly than the concrete. Approximately 20 mm of sheet protrudes beyond the concrete. When built, concrete and steel were on one plane.

Tests were conducted on the operating tank. The lateral surfaces of the ceiling slab well visible in the manhole openings were highly corroded. The loss of concrete ranged from a dozen mm up to more than 20 mm. The folded sheet in the cutting location was also corroded. However, the corrosion rate was lower. Approximately 20 mm of sheet protrudes beyond the corroded concrete (Figure 2).

**Figure 3:**
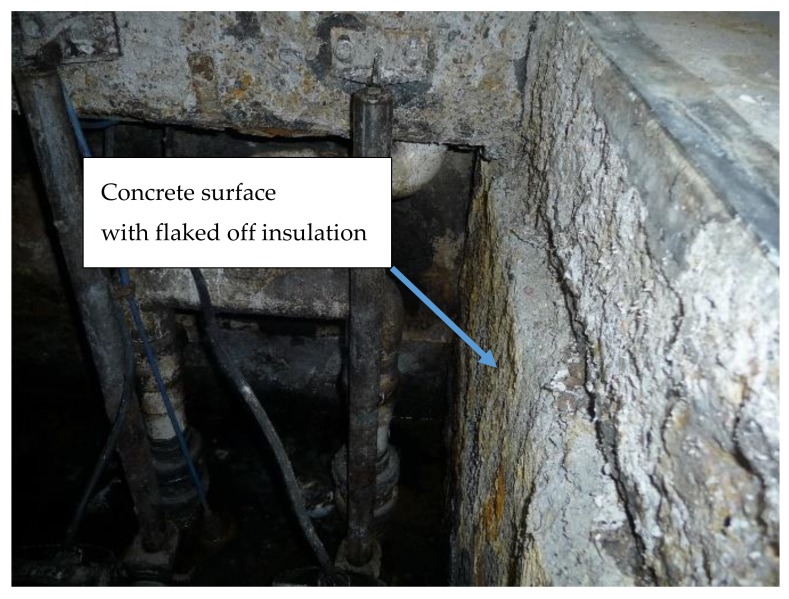
Walls below the ceiling slab were originally covered with insulation but in a considerable area of the walls this insulation had already flaked off.

Such a high degree of concrete corrosion in a zone where it did not have any contact with effluents suggested that in the lower point where effluents were continuously contacting the concrete walls the situation would probably not be better, and might be even far worse. Although the tank walls had some traces of bituminous insulation, large areas lacked this coating because it had flaked off during use (Figure 3).

At the time when the tests were performed the tank was in use and it was necessary to blind the holes after the completed tests. For this reason, in order to perform the measurements, it was decided to make one borehole with a diameter of 103 mm and other boreholes with diameters of 50 mm. The boreholes were made in the direction perpendicular to surfaces of the walls which had been formed in the built-in position (in the vertical direction). Since the boreholes were made at the same level (drilled perpendicularly to the element forming direction), in this case the variability of aggregate in the sample cross-section can be only random. The tests concerning strength distribution in concrete elements conducted by the authors and other researchers indicate the differentiation of strength with respect to the forming direction as a result of segregation of components in the gravitational field of the Earth and draining of water from concrete mix (bleeding) [24,25,26]. Such differentiation does not appear in the horizontal direction appropriate for the taken sample element.

### 2.2. Methods

The compressive strength of concrete samples in their various cross sections were determined with use of the ultrasound method on basis of longitudinal wave velocities [27]. The ultrasonic pulse velocity of a homogeneous solid can be related to its mechanical properties. Theoretical dependencies between ultrasonic wave velocity and elastic modulus and Poisson’s ratio were investigated and described in the literature [28,29]. Based on the theory of elasticity applied to homogeneous and isotropic materials, for the method of testing used by the authors passing wave velocity *C_L_* is directly proportional to the square root of the dynamic modulus of elasticity *E_d_*, and inversely proportional to the square root of its density, ρ, where ν_d_ is the dynamic Poisson’s ratio (5):

*C_L_* = (*E_d_*/*ρ* ∙ (1 − *ν_d_*)/((1 + *ν_d_*) ∙ (1 + 2*ν_d_*)))^1/2^ [km/s] (5)

Concrete is a heterogeneous material, so these assumptions are not strictly valid. High attenuation in concrete limits the ultrasonic pulse velocity method (UPV) to frequencies up to 100 kHz, which means that compressional waves do not interact with most concrete inhomogeneities [30]. Under this condition concrete can be regarded as a homogeneous material [31]. The tests conducted already in the seventies and eighties of the 20^th^ century showed that there is a relationship between ultrasonic wave velocity and concrete strength. The possibility of using the correlation between these values was included both in scientific literature e.g., [28,29,32,33,34,35,36,37,38] as well as in norms e.g., [39,40,41]. In the study [38], Komlos and others compared eight basic methods of determining concrete strength based on measurements of ultrasound velocity. He concluded that the necessary requirement of such tests was to perform calibration of measurements with results of destructive tests, such are also research experiences of the authors [37,42]. The confirmation regarding the possibility of using the measurements of ultrasonic wave velocity to test compressive strength of concrete exposed to sulphate corrosion can be found in scientific literature from the beginning of this century and from the later years [43,44,45].

**Figure 4:**
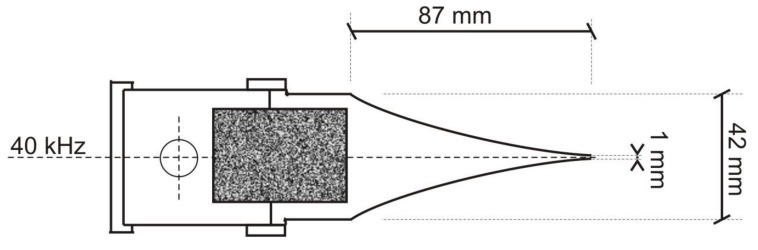
Ultrasonic spot head with the exponential concentrator.

Measurements were performed using ultrasonic point probes of frequency equal to 40 kHz the testing results of which were presented in the study [37]. The structure of the probes is shown in the Figure 4. They were equipped with exponential, half wave concentrators with a length of 87 mm and base width of 42 mm. The diameter of the contact point of the concentrators was 1 mm.

**Figure 5:**
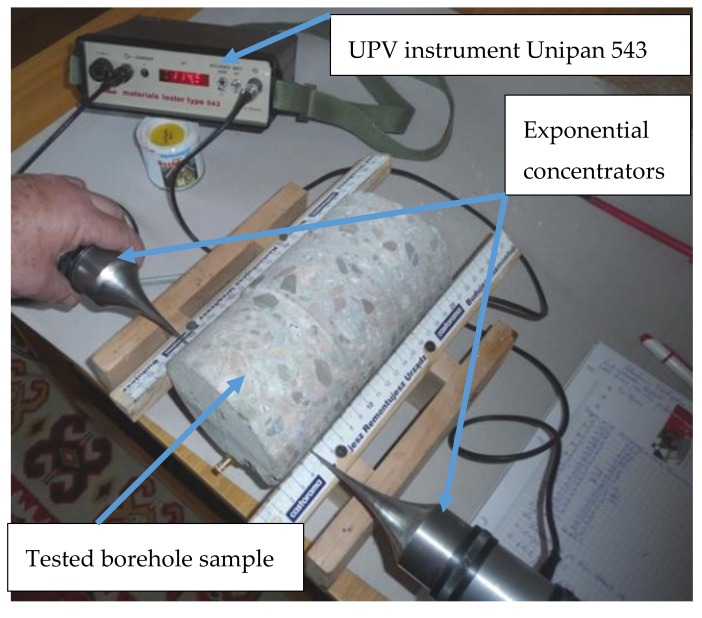
Borehole No. 1 during ultrasound tests.

The tests were performed with a UNIPAN 543 (Zaklady Aparatury Naukowej UNIPAN, Warsaw, Poland) ultrasonic pulse velocity (UPV) test instrument (Figure 5). Probe concentrators were applied from the two opposite sides of the examined concrete cylindrical samples, in planes parallel to the surface of the wall they were bored from. In that way the longitudinal wave velocities were measured.

**Figure 6:**
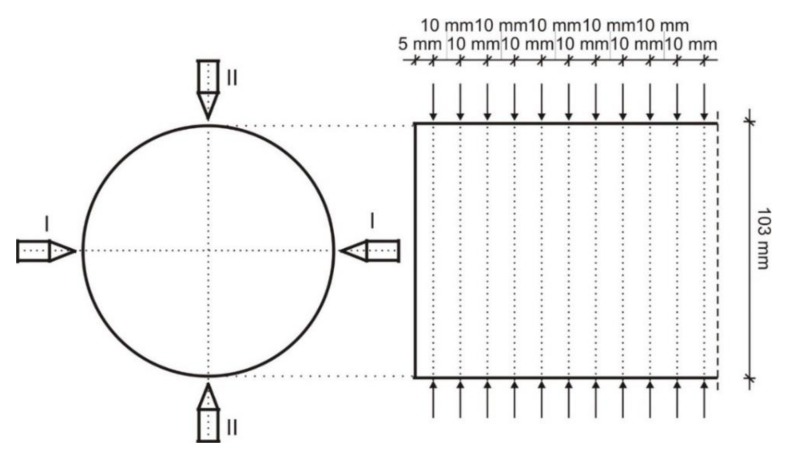
Layout of measuring points and sections on the tested boreholes.

The ultrasound rate was determined in two directions approximately perpendicular to each other, along the diameters, in planes located 10 mm from each other. Only the distance of the first measurement point from the external surface of the wall was 5 mm (Figure 6).

The examined borehole materials were cut into samples of length equal to their diameter. Boreholes were cut thus obtaining samples with ϕ = h = 10.3 cm and ϕ = h = 5.0 cm. The strength of samples with ϕ = h = 10.3 is equivalent to the strength tested on cubic samples, side 15 cm [29,41,42]. The coefficient for calculation of the strength of samples with ϕ = h = 05.0 cm to the strength tested on cubic samples, side 15 cm is 1.08, what has been tested experimentally by Brunarski [29] and by the authors [42] in the expected strength range of the samples. To the ultrasound rate determined in the middle of the height of each sample, destructive strength was assigned as determined on the strength machine as a relation of destructive force *P* [N] to the surface area of cross-section *A* [mm^2^] (6):

*f_c_* = *P/A* [N/mm^2^] (6)

On that basis a hypothetical scaling curve was chosen and the pairs of results were obtained: compressive strength *f_c_* [N/mm^2^]-passing wave velocity *C_L_* [km/s] according to the methodology described in the literature [29,39].

In order to confirm the salt formation in the concrete, a colorimetric semi-quantitative method has been used with the use of Merck test strips. Then, in order to determine the distribution of sulphate salts forming along the ultrasonic measurements, a gravimetric quantitative method has been used [46,47,48,49]. Tested samples of concrete were cut, dried and crushed. In the next step, samples were extracted in one molar hydrochloric acid. As the precipitating agent barium chloride was added to the pre-heated samples extract to precipitate barium sulphate, which was weighed after washing and calcination at 800 °C to constant weight. Determination of the sulphate content in the barium sulphate precipitate was determined on the basis of mass proportions according to the Equation (7):

x = a 96.064/233.400 = a 0.4116 [g](7)

where *a* is a mass of the barium sulphate [g], *x* is a mass of SO_4_^2−^ in the tested sample of material.

## 3. Results

### 3.1. Quantification of Sulphates in Tested Material

Concrete samples were taken from the walls and ceiling in order to determine salt content. It turned out that chlorides were available in the concrete in acceptable quantities (maximum in sample 1–0.085% of weight of the concrete), sulphates in high quantities above 1.2% by weight of the concrete. No nitrates or nitrites were found in the concrete. In the next step quantitative determination of sulfates using gravimetric method has been undertaken. Tests have been performed on the internal surface of the tank walls, and across its thickness, at distances of 20, 50 and 100 mm from the surface under sulphate attack. Quantity of the sulphates has been calculated with use of the Equation (7). The results of the analysis are presented in Table 1.

Performed quantitative analysis of sulphates on the thickness of the tested samples indicates their deep penetration and concentration in the entire cross-section of the walls. The highest concentration of SO_4_^2−^ has been examined in the zone near the inner wall surface (above 1.52%), at a depth of 20 mm SO_4_^2−^ percentage concentration was 1.35%, reaching 0.55% at a depth of 50 mm and 0.31% at a distance of 100 mm from the inner tank wall surface.

### 3.2. Calibration of Ultrasound Pulse Velocity-Compression Strength Curve Based on the Destructive Tests

**Figure 7:**
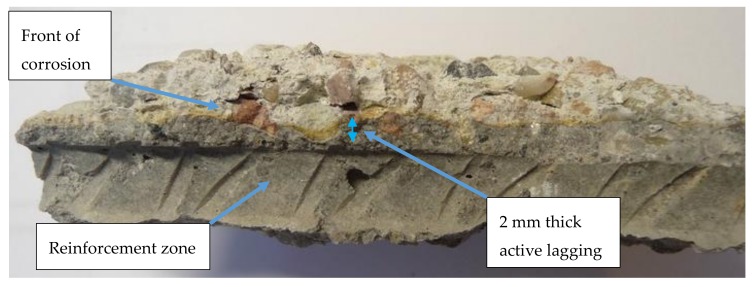
Thickness of non-corroded lagging in borehole No. 1 is only 2 mm.

Since the distribution of compressive strength across the thickness of reinforced concrete structures subject to sulphate corrosion from one side had not been tested so far, the authors performed such measurements using the ultrasonic method. In order to investigate the actual condition of concrete corrosion in the walls, three boreholes were made. The borehole location chosen was below the ceiling slab but in a manner ensuring that effluents did not overflow. The first and second borehole were made at the distance of 50 cm from the top wall edge, and the third borehole at the height of the top wall edge. Borehole No. 1 was of diameter 103 mm, and 2 & 3 diameter 50 mm. It was noted that on borehole No. 1 reinforcing meshes had moved towards the internal surface. For this reason, the thickness of lagging was reduced down to 10 mm and as a result of corrosion in the tested location 8 mm of concrete were missing, only a protective layer 2 mm thick (Figure 7) remained.

After the measurements of ultrasonic pulse velocities of the tested samples they have been cut and tested in uniaxial loading on strength machine. On that base a hypothetical scaling curve with the following Equation (8) was chosen:

*f_c_* = 53.6∙*C_L_* − 122.3 [N/mm^2^] (8)

where *f_c_* is the compressive strength of concrete [N/mm^2^], and *C_L_* is the ultrasound longitudinal wave velocity [km/s]. The results of destructive tests, measured pulse velocities and strength values calculated with use of Equation (8) are presented in Table 2.

In this way, scaling curves established hypothetically were used to convert the rate of ultrasound wave in the given cross-section at the borehole height into concrete compression strength in this cross-section.

### 3.3. Testing the Strength of Concrete across the Tank Wall Thickness

Passing times *t* [μs], calculated wave velocities *C_L_* [km/s] and compressive strengths *f_c_* [N/mm^2^] in planes parallel to the surface of the boreholes are presented in Table 3. Results are presented starting with the ordinal number 1 (5 mm from the external side of the tested wall) in the direction of the internal side of the examined tank.

**Figure 8:**
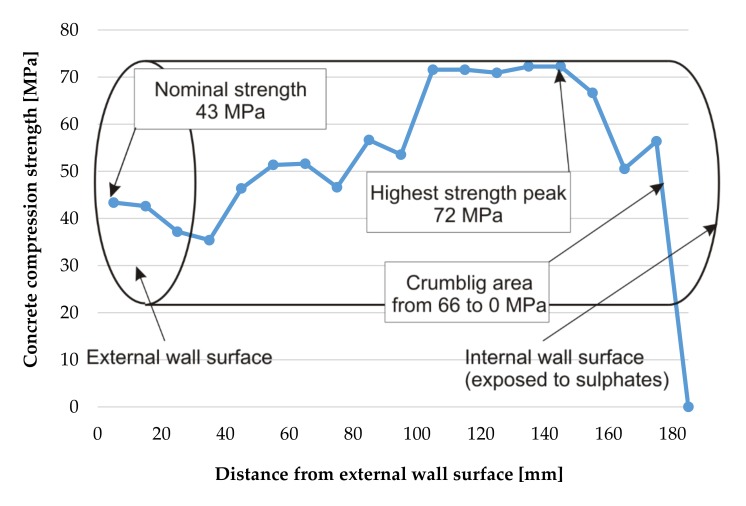
Change of concrete strength across the tank wall thickness in borehole No. 1.

The dependencies of compressive strength as a function of depth for the borehole No. 1 are shown in Figure 8.

Similar tests were performed on boreholes No. 2 & 3 which broke and reinforcement was not cut, hence their length is less than the thickness of the tank wall. The results of tests performed on borehole No. 2 are shown in Table 4.

**Figure 9:**
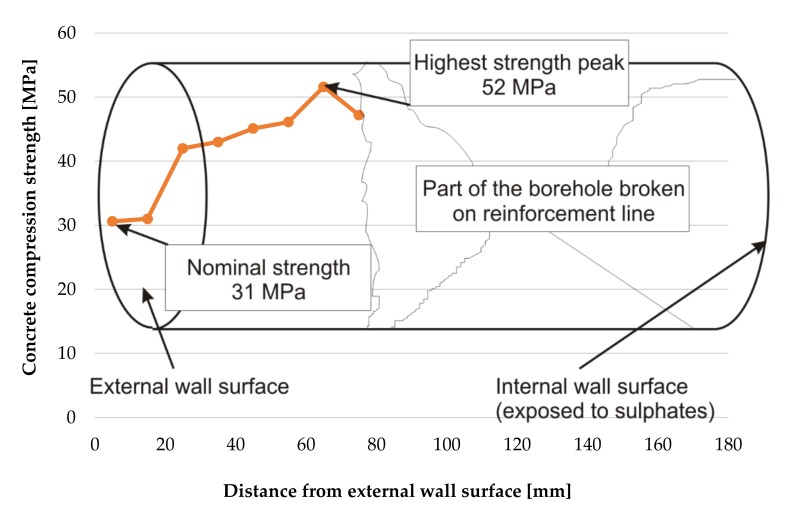
Change of concrete strength across the tank wall tested on borehole No. 2.

Core no 2 was broken at the reinforcement mesh at a depth of 75 mm from the external surface of the wall. On the tested (not damaged) fragment of the core the growing dependency of strength as a function of depth was established. The results from Table 3 are depicted in Figure 9.

Results of tests performed on borehole No. 3 which was also broken in the middle of the tested section are shown in Table 5.

**Figure 10:**
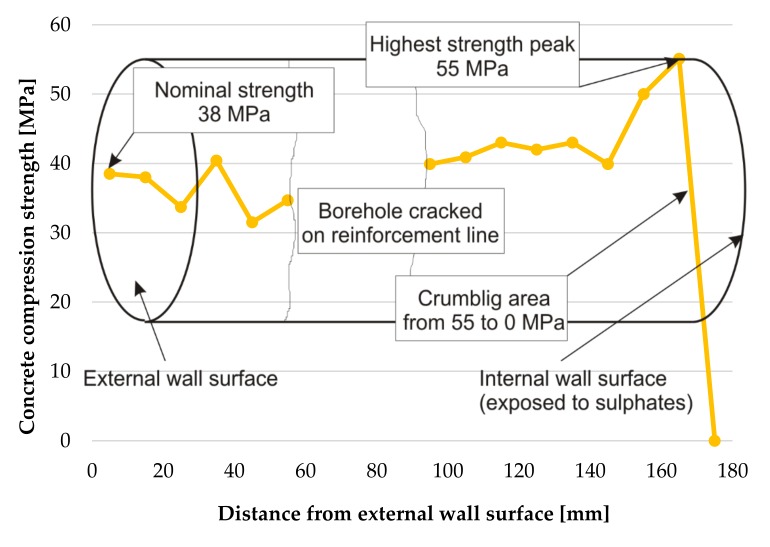
Change of concrete strength across the tank wall tested on borehole No. 3.

Core No. 3 was cracked at the reinforcement at a distance of 60 to 80 mm from the external surface of the wall. For this reason the tests were not performed in this part of the core. On the tested (not damaged) fragment of the core the growing dependency of strength as a function of depth was established. The results from Table 2 are depicted in Figure 10.

**Figure 11:**
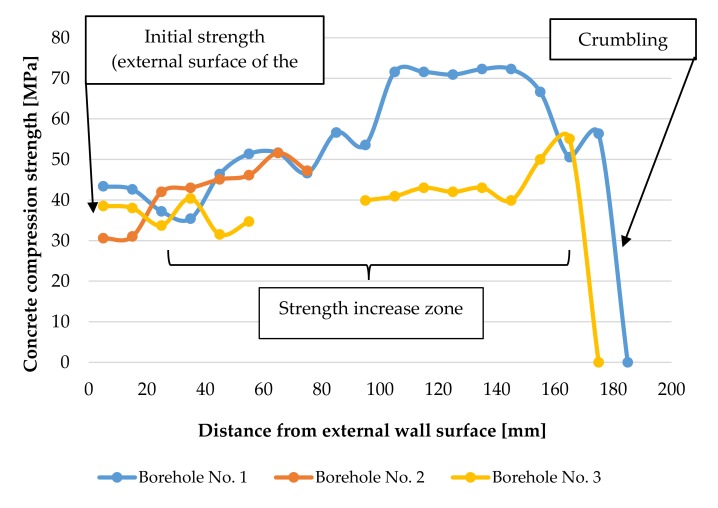
Change of concrete strength across the tank wall thickness in three boreholes.

The method of measuring ultrasonic wave velocity using spot heads presented in this paper allowed determination of the distribution of strength in the cross-sections of reinforced concrete elements exposed to sulphate corrosion (with heterogeneous mechanical properties). The observations presented in this study show that the compressive strength of concrete subjected to a gaseous, aggressive, sulphuric environment is variable across its thickness. Concrete strength is variable across the wall thickness, however initially it was expected that by moving towards the tank interior, the strength would decrease, and from all boreholes the growing dependence was obtained, what has been shown in Figure 11.

Values of compressive strength of the samples taken from the tank walls show an increase in strength from 30–43 N/mm^2^ in the cross sections near the external, unexposed wall layers to 55 and 72 N/mm^2^ in the internal (exposed) cross sections of the walls. For the tested samples, this gives an increase in strength of 44% to 83% from its initial strength measured in the cross sections near the unexposed side of the structure. Sulphuric acid ions react with the cement compounds and the formation of gypsum leads to an increase in volume [17,18,50]. No destructive expansion of concrete takes place at this stage, and a reasonable conclusion can be drawn that the filling of pores and spaces in concrete by the calcium sulphate dihydrate causes a significant increase in the strength of the concrete, as has been shown in Figure 11. The compressive strength of the concrete samples decreases suddenly in the inner cross sections of the walls, when the salt crystallization pressure exceeds concrete tensile strength. In this stage of corrosion gypsum stone reacts with tricalcium aluminates (C3A) and hydrated calcium sulfoaluminate (monosulphate) and forms the final chemical product Candlot’s salt what is associated with spalling and cracking in the surface zone of tested elements, and has been described in [51,52,53]. The completed tests confirmed that the drop of compressive strength took place only near internal cross-sections of the tested samples, within a distance not greater than 10 mm from their exposed surface. In the future, it is planned to carry out measurements of ultrasonic wave velocity and strength distribution in the tank walls at the height at which they are immersed in the corrosive substance and to compare them with the results presented in this article.

## 4. Conclusions

Based on the case study and its analysis presented in this article, the following conclusions can be made:

- Ultrasound testing methodology allowed determination of the distribution of strength as a function of depth of concrete elements under sulphate attack.
- The compressive strength of the concrete exposed to sulphate attack from one side is variable across its depth.
- The experimentally tested distribution of compressive strength at the depth of the elements showed an upward trend in the entire cross section towards the surface subject to corrosion.
- A decrease of strength appears only in the destroyed, crumbled zone of the concrete structure. The destroyed zone of tested elements did not exceed a depth of 10 mm from the surface exposed to sulphates attack.
- In the presented research, the difference in concrete strength between cross sections near the exposed and unexposed sides varied from an increase of 44% (borehole No. 3) to 83% (borehole No. 1).
- The performed tests indicate that gases may be a more corrosive environment, especially with high humidity, than liquids, therefore the coefficients of diffusion resistance or other permeability parameters, e.g., g/m^2^/24 hours, are important parameters characterizing anticorrosive coatings. Chemical resistance to various acids, alkalis or other compounds is tested for the specific aggressive compound at a given level.
- In the tested tank, sulphur-containing gas (hydrogen sulphide) easily penetrated through the thin bituminic layer, based on the measurements at a thickness of 0.97 mm, and in contact with cement and lime formed sulphates, considerable quantities of which were found in the concrete.
- Since the lagging thickness had decreased already down to 2 mm, danger exists not only for the concrete but also for steel. The rate of concrete corrosion in the tank is probably influenced by the concentration of the gases above the liquid. If the tank had a properly built gravity ventilation, the concrete damage process would be much slower, because relative air humidity in the tank would also be much lower with effectively running ventilation.

## Figures and Tables

**Table 1:** Results of the gravimetric quantification of sulphates across the thickness of tested concrete elements.

Distance from Internal Wall Surface [mm]	Concrete Sample Mass [g]	Mass of BaSO_4_ (a) [g]	Mass of SO_4_^2−^ (x) [g]	SO_4_^2−^ [% by Sample Weight]	SO_4_^2−^ Mean Value [%]
0	10.2327	0.3796	0.1563	1.527	1.524
	10.1276	0.3873	0.1594	1.574	
	10.3214	0.3691	0.1519	1.472	
20	9.9885	0.3211	0.1321	1.323	1.345
	10.1445	0.3436	0.1414	1.394	
	10.3417	0.3312	0.1363	1.318	
50	10.1424	0.1163	0.0479	0.472	0.548
	9.9672	0.1480	0.0609	0.611	
	10.1228	0.1382	0.0569	0.562	
100	9.8276	0.0766	0.0315	0.321	0.315
	10.1412	0.1015	0.0418	0.412	
	10.3429	0.0533	0.0219	0.212	

**Table 2:** Results of uniaxial destructive tests, measured pulse velocities and strength values calculated with use of the chosen hypothetical scaling curve.

Sample No.	Core Size [cm × cm]	Ultrasound Longitudinal Wave Velocity *C_L_* [km/s]	f_c,Ø_-f_c,cube_ Conversion Factor	Compression Strength [MPa]		
				Destructive Test		*f_c_* from Equation (6)
				f_c, Ø_	f_c, cube_	
1	10.3 × 10.3	3.63	1.00	68.92	68.92	72.27
2	10.3 × 10.3	2.94		37.55	37.55	35.28
3	5.0 × 5.0	3.08	1.08	41.94	45.3	42.79
4	5.0 × 5.0	2.91		32.66	35.27	33.68
5 6	5.0 × 5.0 5.0 × 5.0	3.14 3.42		40.90 59.62	44.17 87.14	46.00 61.01
Mean value	-	3.19	-	46.20	48.47	48.51

**Table 3:** Results of concrete compression strength test in borehole No. 1.

Ordinal Number	Ultrasound Netto Passing Time in Direction I-I *t_n I-I_* [μs]	Ultrasound Netto Passing Time in Direction II-II *t_n II-II_* [μs]	Mean Ultrasound Netto Passing Time *t_n_* [μs]	Ultrasound Longitudinal Wave Velocity *C_L_* [km/s]	Concrete Compression Strength *f_c_* [N/mm^2^]
1	33.00	34.30	33.65	3.09	43.36
2	33.40	34.20	33.80	3.08	42.62
3	34.20	35.70	34.95	2.98	37.20
4	35.30	35.40	35.35	2.94	35.39
5	33.50	32.60	33.05	3.15	46.37
6	33.00	31.20	32.10	3.24	51.36
7	30.60	33.50	32.05	3.25	51.63
8	32.20	33.80	33.00	3.15	46.62
9	30.30	32.00	31.15	3.34	56.65
10	29.60	33.80	31.70	3.28	53.55
11	28.40	29.10	28.75	3.62	71.59
12	28.50	29.00	28.75	3.62	71.59
13	28.70	29.00	28.85	3.61	70.92
14	28.70	28.60	28.65	3.63	72.27
15	28.70	28.60	28.65	3.63	72.27
16	28.70	30.30	29.50	3.53	66.66
17	32.20	32.30	32.25	3.23	50.55
18	31.40	31.00	31.20	3.33	56.37
Mean (1–18)	31.13	31.91	31.52	3.32	55.39
19	-	-	-	-	0.00

**Table 4:** Results of concrete compression strength test in borehole No. 2.

Ordinal Number	Ultrasound Passing Time, Direction I-I *t_n I-I_* [μs]	Ultrasound Passing Time, Direction II-II *t_n II-II_* [μs]	Mean Ultrasound Passing Time *t_n_* [μs]	Ultrasound Longitudinal Wave Velocity *C_L_* [km/s]	Concrete Compression Strength *f_c_* [N/mm^2^]
**1**	17.70	17.00	17.35	2.85	30.57
**2**	18.50	16.10	17.30	2.86	31.05
**3**	16.20	16.10	16.15	3.06	41.93
**4**	16.10	16.00	16.05	3.08	42.95
**5**	16.40	15.30	15.85	3.12	45.04
**6**	15.80	15.70	15.75	3.14	46.11
**7**	15.00	15.50	15.25	3.25	51.63
**8**	15.80	15.50	15.65	3.16	47.18
Mean (1–8)	16.44	15.90	16.17	3.07	42.06

**Table 5:** Results of concrete compression strength test in borehole No. 3.

Ordinal Number	Ultrasound Passing Time, Direction I-I *t_n I-I_* [μs]	Ultrasound Passing Time, Direction II-II *t_n II-II_* [μs]	Mean Ultrasound Passing Time *t_n_* [μs]	Ultrasound Longitudinal Wave Velocity *C_L_* [km/s]	Concrete Compression Strength *f_c_* [N/mm^2^]
1	16.00	17.00	16.50	3.00	38.5
2	16.30	16.80	16.55	2.99	38.0
3	16.60	17.40	17.00	2.91	33.7
4	16.00	16.60	16.30	3.04	40.4
5	16.90	17.60	17.25	2.87	31.5
6	16.90	16.90	16.90	2.93	34.7
7–9	-	-	-	-	
10	16.30	16.40	16.35	3.03	39.9
11	16.20	16.30	16.25	3.05	40.9
12	15.60	16.50	16.05	3.08	43.0
13	15.70	16.60	16.15	3.06	42.0
14	15.30	16.80	16.05	3.08	43.0
15	16.30	16.40	16.35	3.03	39.9
16	14.80	16.00	15.40	3.21	50.0
17	15.10	14.80	14.95	3.31	55.1
Mean (1–6,10–17)	16.00	16.58	16.29	3.04	40.8
18	-	-	-	-	0.00
